# Wide Riparian Zones Inhibited Trace Element Loss in Mining Wastelands by Reducing Surface Runoff and Trace Elements in Sediment

**DOI:** 10.3390/toxics12040279

**Published:** 2024-04-11

**Authors:** Jiangdi Deng, Zuran Li, Bo Li, Cui Xu, Lei Wang, Yuan Li

**Affiliations:** 1 Faculty of Animal Science and Technology, Yunnan Agricultural University, Kunming 650201, China; dengjd.cn@gmail.com (J.D.); cuixu0415@126.com (C.X.); 2 College of Horticulture and Landscape, Yunnan Agricultural University, Kunming 650201, China; lizuran@foxmail.com; 3 College of Resources and Environment, Yunnan Agricultural University, Kunming 650201, China; libo@ynau.edu.cn (B.L.); wanglei.ko@foxmail.com (L.W.)

**Keywords:** mining wasteland, riparian zone, width, trace element loss, field experiment

## Abstract

The diffusion of trace elements in mining wastelands has attracted widespread attention in recent years. Vegetation restoration is an effective measure for controlling the surface migration of trace elements. However, there is no field evidence of the effective riparian zone width in mining wastelands. Three widths (5 m, 7.5 m, and 10 m) of *Rhododendron simsii*/*Lolium perenne* L. riparian zones were constructed in lead–zinc mining wastelands to investigate the loss of soil, cadmium (Cd), copper (Cu), arsenic (As), lead (Pb), and zinc (Zn). Asbestos tiles were used to cut off connections between adjacent plots to avoid hydrological interference. Plastic pipes and containers were used to collect runoff water. Results showed that more than 90% of trace elements were lost in sediment during low coverage and heavy rainfall periods. Compared with the 5 m riparian zone, the total trace element loss was reduced by 69–85% during the whole observation period in the 10 m riparian zone and by 86–99% during heavy rain periods in the 10 m riparian zone, which was due to reduction in runoff and concentrations of sediment and trace elements in the 10 m riparian zone. Indirect negative effects of riparian zone width on trace element loss through runoff and sediment concentration were found. These results indicated that the wide riparian zone promoted water infiltration, filtered soil particles, and reduced soil erosion and trace element loss. Riparian zones can be used as environmental management measures after mining areas are closed to reduce the spread of environmental risks in mining wastelands, although the long-term effects remain to be determined.

## 1. Introduction

Trace elements are released into the soil, water, and atmosphere during mineral mining and smelting, disrupting the biogeochemical cycle of these elements. Heavy metals and metalloids such as Cr, Ni, Cu, Zn, Cd, Pb, Hg, and As pose a significant risk to the environment. Over time, the pollution levels and associated risks of these elements in the environment accumulate [1]. The accumulation of trace elements in the soil in mining areas is influenced by altitude, slope, landscape, wind, rainfall, and water flow. Water flow transports large quantities of trace elements, while wind transports trace elements over long distances. The spread of trace element pollution causes soil degradation and landscape degradation [2,3]. Following mine closures, trace elements are redistributed in the form of sediments over short periods of time, increasing the geo-accumulation index of potentially toxic trace elements in farmland and rivers and increasing the risk of human exposure [4]. Riparian zone vegetation restoration would change the distribution of trace elements in low-altitude areas, which is important for reducing downstream environmental risks [5].

The riparian zone is an important riverside landscape corridor with the characteristics of high productivity and high biodiversity. A long and narrow vegetation belt connects the river and the embankment, providing space for the migration and distribution of water, sediment, nutrients, animals, and plants on the riverbank [6]. The vegetation barrier in the riparian zone effectively controls non-point source pollution by intercepting runoff and filtering suspended solids. The plants in the riparian zone play a crucial role in adsorbing, absorbing, and transforming pollutants, as well as reducing erosion [7,8,9]. However, the plant community species diversity and vegetation coverage in the natural restoration area in mining wastelands are lower than those in artificial restoration sites [10]. Complex spatial structures and species configurations of the vegetation within the riparian zone have proven to be effective at intercepting pollutants [11,12]. Additionally, vegetation coverage significantly reduces the erosion intensity and volume in riparian zones [13]. However, the water and soil conservation functions of the riparian zone are severely degraded in many managed areas, particularly in areas exposed to seasonal water erosion following mine closure.

Runoff resulting from terrain and rainfall transport trace elements into rivers, which is most serious during the rainy season. Soil and trace elements in sediment loss exhibit a similar spatial trend [14,15,16]. Plants are poisoned by trace elements, leading to reduced vegetation coverage and increased soil erosion. The sediment erosion rate decreases with the restoration of vegetation. Riparian zones with complex spatial structures intercept rainfall, protect soil from splash erosion, increase infiltration, and reduce sediment erosion [17]. Wide riparian zones exhibit extended hydraulic retention times and increased infiltration of runoff. Sediment and trace elements in sediment are intercepted in the grass riparian zone due to its high stem density. As a result, the decrease in runoff rate leads to a reduction in sediment and pollutant loss [18,19]. Increasing the coverage of the riparian zone resulted in a reduction in trace elements in runoff [20]. Research has shown that increasing riparian zone width is important in reducing pollutant concentration [21]. Model calculation results suggested that trace elements in sediment and dissolved pollutants need a riparian zone width of 10 m and 50–70 m, respectively, with an intercept rate of 90% [22]. Although studies have proposed effective ecological restoration measures for different types of mines, the impact of riparian zone widths on trace element loss in mining wasteland riverbanks remains unclear.

The Lanping lead–zinc mining area is situated in southwest China and is one of the largest lead–zinc deposits in Asia. Rainfall is concentrated from May to October each year, with trace elements from abandoned mining wasteland deposited in the ditch sediment. The degree of trace element pollution in these rivers is affected by seasonal rainfall, posing significant ecological risks [4,23]. In Lanping, several models of vegetation restoration and agriculture have been applied. However, there is insufficient evidence to determine the effective width of the riparian zone in mining wastelands for controlling trace element loss. Therefore, three widths (5 m, 7.5 m, and 10 m) of *Rhododendron simsii* Planch./*Lolium perenne* L. riparian zones were constructed on the riverbank of the abandoned lead–zinc mine in Lanping. This study examined the characteristics of vegetation restoration and trace element loss in these riparian zones under natural rainfall to study the effects of riparian zone width and vegetation coverage on runoff, sediment, and trace element loss concentration and loss volume and determined the effective width of trace elements reduction in the riparian zone in mining wastelands.

## 2. Materials and Methods

The experimental field is located 5 km east of Lanping County in Yunnan Province, Southwest China, at 26°46′ N and 99°47′ E and an altitude of 2880 m above sea level. The area is a subtropical alpine forest with a low latitude mountain monsoon climate, an annual average temperature of 10.7 °C, and an annual average rainfall of 1002 mm. The basic chemical properties of the soil are as follows: The soil pH is 7.21; the soil organic matter content is 8.45 g kg^−1^; the total nitrogen, phosphorus, and potassium contents are 1.58 g kg^−1^, 9.38 g kg^−1^, and 1.49 g kg^−1^, respectively; and the alkali-hydrolysable nitrogen, available phosphorus, and available potassium contents are 20.13 mg kg^−1^, 9.52 mg kg^−1^, and 45.38 mg kg^−1^, respectively. The contents of Cd, Pb, Cu, Zn, and As are 215.37 mg kg^−1^, 20193 mg kg^−1^, 167.37 mg kg^−1^, 34412 mg kg^−1^, and 446.63 mg kg^−1^, respectively [24].

**Figure 1:**
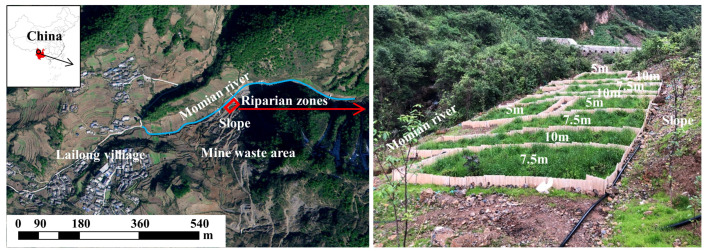
Experimental site and design of riparian zones.

The experiment was carried out during the rainy season. Riparian zones were constructed on the banks of the Momian River in April 2021 (Figure 1). The *R. simsii*/*L. perenne* intercropping pattern was adopted, with *R. simsii* planted at spacings of 0.8 × 0.6 m and *L. perenne* sown at 180 kg ha^−1^. Each riparian zone with a water catchment area of 25 m^2^ was constructed along the slope, and the widths of the buffer zone were 5 m, 7.5 m, and 10 m (the corresponding horizontal lengths were 5 m, 3.3 m, and 2.5 m). Three replicates were set for each width treatment. Nine plots were randomly arranged at a slope of 15%. Asbestos tiles (0.3 m underground and 0.7 m above ground) were used to separate the small areas to cut off connections between adjacent plots and avoid hydrological interference. A 100 L plastic collection container was placed in each plot and connected to the plot through plastic pipes to collect surface runoff. Urea (total nitrogen ≥ 46%), potassium sulfate ≥ 52%), organic fertilizer (organic matter content ≥ 50%), and phosphate fertilizer (superphosphate ≥ 16%) were applied before sowing.

The experimental observation period was from April to September. Six daily rainfall samples and one 60-min continuous rainfall sample were collected. Rainfall amount and frequency were recorded at the weather station. Runoff volume and coverage for each plot were recorded. One thousand milliliters of mixed runoff water were collected per rainfall. Plants within 1 m^2^ were collected on September 6. Plant samples were cut into shoot and root parts, washed with deionized water, dried at 105 °C for 30 min, and dried at 80 °C to a constant weight. Plants were ground into powder with a mortar and pestle and sieved through a 1 mm nylon sieve for the determination of trace elements content.

One hundred milliliters of well-mixed runoff sample were suctioned and filtered through a dried 0.45 μm water-based microporous filter membrane and dried at 105 °C to a constant weight. The filtrate was collected and preserved at 4 °C. The sediment content in the runoff was calculated as follows:

$$C=(w_{1}-w_{2})\times 10^{6}/V$$

where C is the sediment content in the runoff, mg L^−1^; w_1_ is the weight of the suspended substance, filter membrane, and weighing bottle, g; w_2_ is the weight of the filter membrane and weighing bottle, g; V is the volume of the runoff water sample, mL.

Plant samples (0.5 g) were placed in PTFE tubes and soaked with 5 mL of nitric acid for 8 h. Then, two milliliters of hydrogen peroxide were added to the PTFE. The PTFE was sealed and digested at 140 °C for 4 h. Next, the digested solution was filtered and diluted to 50 mL with deionized water. The filtrate and filter membrane with suspended matter were placed in a conical bottle and soaked with 1 mL of nitric acid, 3 mL of hydrochloric acid, and 2 mL of perchloric acid. The mixture was digested on an electric heating plate to obtain a transparent solution. The digested solution was then diluted to 50 mL with deionized water. The contents of Cd, Pb, Cu, and Zn in solution were determined using an atomic absorption spectrometer (ICE 3300, Thermo Fisher, Karlsruhe, Germany). The contents of As in solution were determined using an atomic fluorescence spectrometer (LC-AFS9600, Haiguang, Beijing, China). Cd, Cu, As, Pb, and Zn standard solutions (1000 µg mL^−1^) were used as reference materials (GSB 04-1721-2004, GSB 04-1725-2004, GSB 04-1714-2004, GSB 04-1742-2004 and GSB 04-1761-2004, General Research Institute for Nonferrous Metals, Beijing, China). Recovered percentages were 93–106% for Cd, Cu, As, Pb, and Zn. A blank test was conducted at the same time.

Soil trace elements were mainly lost in sediment or dissolved form. The percentage of trace elements in sediment (P_TRs_) was used to represent the ratio of trace elements in sediment to total trace elements. P_HMs_ indicated the output form (sediment or dissolved) of trace elements from the riparian zone.

$$P_{\mathrm{TRs}}={\mathrm{TRs}}_{\mathrm{sediment}}/{\mathrm{TRs}}_{\mathrm{runoff}}$$

P_TRs_ is the ratio of trace elements in sediment to total trace elements; TRs_sediment_ is the loss of trace elements in sediment; TRs_runoff_ is the total amount of trace elements in runoff; HMs include Cd, Cu, As, Pb, and Zn.

Origin 8.0 was used for drawing. The SPSS 20.0 statistical software was used for conducting one-way ANOVA, Duncan test, and two-way ANOVA. To determine the relationship between the loss form of trace elements and environmental factors, the mantel test was implemented in the vegan package in R. Principal component analysis (PCA) was used to transform the data set (Cd, Cu, As, Pb, and Zn) into a set of comprehensive principal components as a comprehensive indicator of trace element loss [25]. Structural equation modeling (SEM) was performed using the lavaan package in R to analyze ways to explain the impact of trace element loss in the riparian zone, to evaluate the direct and indirect factors affecting trace elements output, and to calculate the standardized total effect of each factor (direct effect adds up to indirect effect) [26].

## 3. Results

### 3.1. Rainfall and Riparian Zone Characteristics

The total rainfall recorded during the observation period (from April to September) was 405.7 mm. Runoffs were collected and analyzed for trace element loss characteristics. After the rainfall event on July 2, the moisture content of the surface soil approached saturation and the ryegrass seeds germinated. After 65 days, the average coverage of the riparian zone increased from 40% to 95% (Figure S1).

### 3.2. Runoff, Sediment, and Trace Element Loss

**Figure 2:**
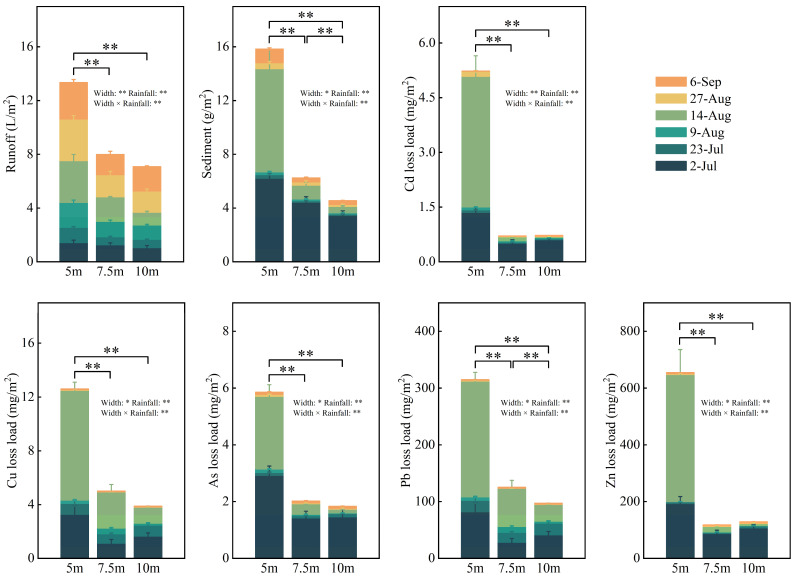
Runoff, sediment, and trace element loss in riparian zones. Note: “*” and “**” mean significant (*p* < 0.05) and very significant (*p* < 0.01) differences between different riparian zone widths, respectively. “*” and “**” after width and rainfall mean that there was an interaction between the buffer zone width and rainfall on the sediment and trace element loss, *p* < 0.05 and *p* < 0.01.

Runoff was neutral and slightly alkaline (Figure S2). Runoff and sediment yield accounted for 11–16% and 39–76% of the total during periods of low vegetation coverage and 14–23% and 10–49% during heavy rainfall periods (Figure 2), respectively. Compared with the 5 m riparian zone, runoff yield in the 7.5 m and 10 m riparian zones reduced by 40% and 47%, respectively, while sediment yield reduced by 61% and 71%, respectively. Total runoff and sediment yield decreased with the extension of the riparian zone width in this experimental plot.

Trace elements in the experimental area were mainly exported during low vegetation coverage and heavy rainfall periods (Figure 2). Trace elements in sediment accounted for 94–99% of the total loss. The loss of trace elements at 5 m, 7.5 m, and 10 m accounted for 26–50%, 23–74%, and 43–84%, respectively, of the total loss during low vegetation coverage periods, and 44–69%, 13–54%, and 4–30%, respectively, during heavy rainfall periods. Trace elements were mainly lost during heavy rainfall periods after coverage increased. Trace element loss significantly decreased with the extension of riparian zone width during heavy rainfall periods in this experimental plot. The two-way ANOVA showed that runoff, sediment, and trace element loss were affected by the interaction between rainfall and riparian zone width.

### 3.3. Sediment and Trace Element Concentrations in Runoff

**Figure 3:**
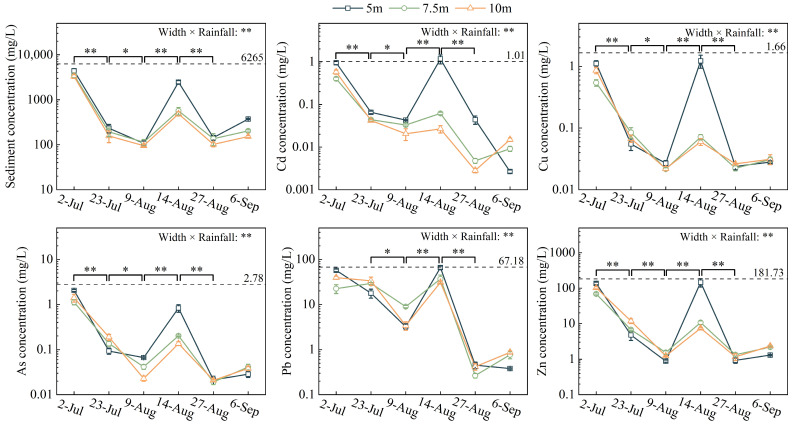
Concentrations of sediment and trace elements in surface runoff. Note: The dotted lines indicate the concentrations of sediment and trace elements in surface runoff samples collected from bare land in the experimental field on 12 August 2020. “*” and “**” above the curve mean significant (*p* < 0.05) and very significant (*p* < 0.01) differences in sediment and trace element concentrations between the two periods, respectively. “**” after “width × rainfall” mean that there was an interaction between the buffer zone width and rainfall on the concentrations of sediment and trace elements, *p* < 0.01.

Sediment concentration and trace element loss exhibited a decreasing stage, an increasing stage, and a decreasing stage (Figure 3). Compared to the background value, the concentrations of sediment and trace elements decreased by 94–98% and 98–99%. Sediment concentration and trace element concentrations increased in all widths of the riparian zone during heavy rainfall periods. Under heavy rainfall, sediment concentration in the 10 m riparian zone reduced by 80% compared with the 5 m riparian zone, while trace element concentrations reduced by 53–98%. The impact of non-heavy rainfall periods on sediment and trace element loss decreased with the increase in vegetation coverage in the riparian zone. The two-way ANOVA showed that sediment and trace element concentrations were affected by the interaction between rainfall and riparian zone width.

**Figure 4:**
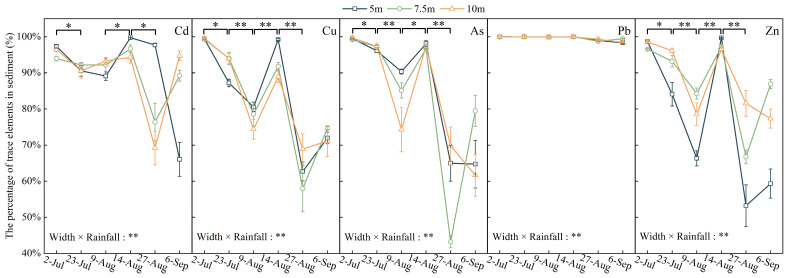
The percentage of trace elements in sediment. Note: “*” and “**” above the curve mean significant (*p* < 0.05) and very significant (*p* < 0.01) differences in the percentage of trace elements in sediments between the two periods, respectively. “**” after width and rainfall mean that there was an interaction between the buffer zone width and rainfall on the loss form of trace elements, *p* < 0.05 and *p* < 0.01.

The P_HMs_ of Cd, Cu, Zn, and As exhibited a decreasing stage, an increasing stage, and a decreasing stage (Figure 4). At the end of the observation, the P_HMs_ of Cd, Cu, As, and Zn had decreased by 13–30% compared with the initial observation. The percentage of Pb in the sediment was stable. The average P_HMs_ increased during the heavy rainfall periods, exceeding 90%. The differences in the P_HMs_ of Cd, As, and Zn between the three widths of riparian zones reduced during the heavy rainfall periods. The two-way ANOVA showed that P_HMs_ was affected by the interaction between rainfall and riparian zone width.

### 3.4. Dynamic Variation Trends in Runoff, Sediment, and Trace Elements in the Riparian Zone

**Figure 5:**
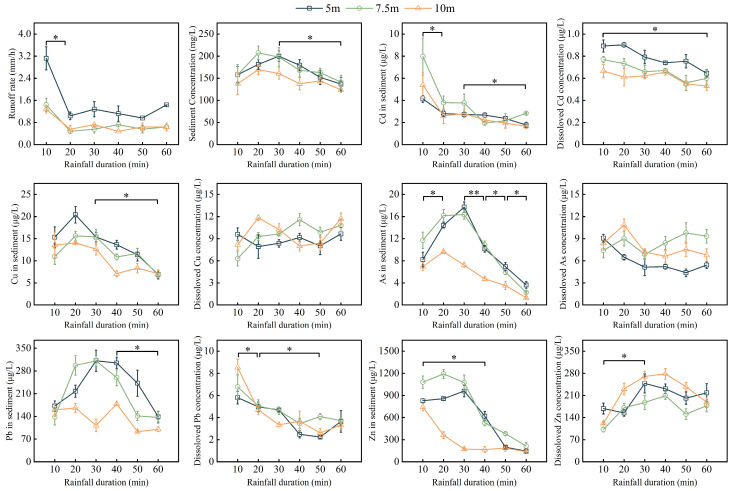
Erosion characteristics of trace elements during the 60-min observation period. Note: “*” and “**” above the curve mean significant (*p* < 0.05) and very significant (*p* < 0.01) differences in the concentrations of trace elements, respectively.

The process of soil erosion and trace elements export in the riparian zone under a natural rainfall intensity of 5.5 mm h^−1^ was observed. Results showed that the runoff generation time was consistent. Compared with the runoff rate at 0–10 min, the runoff rate at 10–60 min significantly decreased (*p* < 0.05) (Figure 5). The concentrations of sediment, Cd, Cu, and As in sediment significantly decreased at 30–60 min (*p* < 0.05). The concentrations of dissolved Pb, Pb, and Zn in sediment significantly decreased during the observation period (*p* < 0.05). The concentration of dissolved Zn increased in 30–60 min (*p* < 0.05). Compared with the 5 m riparian zone, the concentrations of Cu, As, Pb, and Zn in sediment and the concentration of dissolved Cd significantly decreased in the 10 m riparian zone (*p* < 0.05), while the concentration of dissolved As significantly increased in the 10 m riparian zone (*p* < 0.05).

### 3.5. The Phytostabilization of Plants for Trace Elements

Phytoextraction is an important remediation measure for soils contaminated with trace elements. The riparian zone width had no effect on plant biomass or trace element accumulation (*p* > 0.05). Compared to *R. simsii*, *L. perenne* had 329% higher biomass (*p* < 0.05) and 172–821% higher trace elements accumulation (*p* < 0.05) in the shoot (Table 1). The amount of Cd, As, Pb, and Zn accumulated in plants was significantly lower than the loss in the 5 m riparian zone (Figure 3) and significantly higher than the loss in the 7.5 m and 10 m riparian zones (*p* < 0.05).

### 3.6. Correlation Analysis

**Figure 6:**
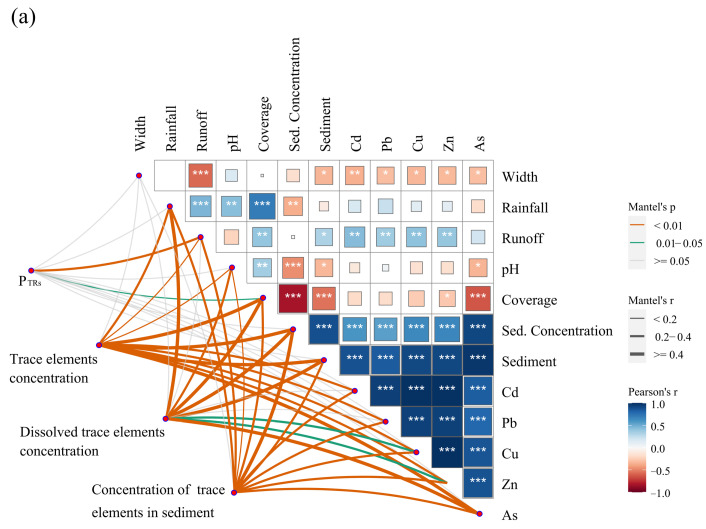

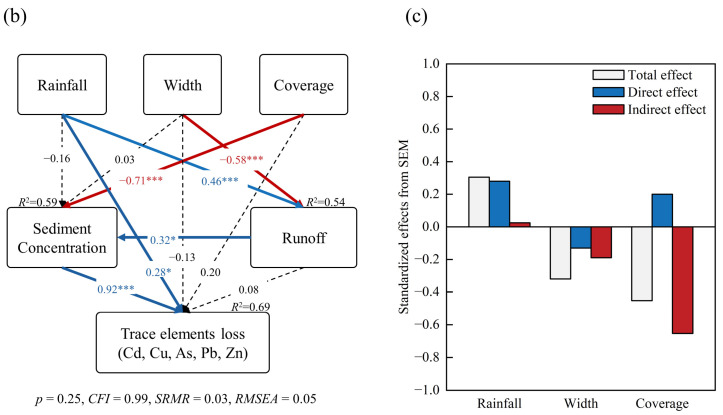
Relationships between rainfall, width, runoff, sediment, and trace element loss. Note: (**a**) The color gradient represents Pearson’s correlation coefficient. Mantel test was used to analyze the correlation between environmental factors and trace element loss characteristics. (**b**) The effects of rainfall, riparian zone width, and vegetation coverage on trace element loss were assessed using structural equation modeling (SEM). A multivariate indicator of trace element loss was generated using principal component analysis (PCA) (Figure S3), including for Cd, Cu, As, Pb, and Zn. The first principal component (PC1, 92.5%) was used for SEM analysis. The dotted lines indicate non-significant normalized path coefficients (*p* > 0.05), and the solid red and blue lines indicate significant negative and positive normalized path coefficients, respectively. The percentage close to the variable represents the variance (R^2^) explained by the model. “*”, “**” and “***” mean *p* < 0.05, 0.001 < *p* < 0.01, and *p* < 0.001, respectively. (**c**) The standardized total effect of each factor in SEM is displayed in the bar chart.

Pearson correlation analysis and the mantel test were performed using the P_TRs_, trace element concentrations, rainfall, riparian zone coverage and width, and sediment and trace element loss (Figure 6). Runoff, sediment, Cd, Cu, As, Pb, and Zn loss were significantly negatively correlated with riparian zone width. Sediment concentration and yield were significantly negatively correlated with riparian zone coverage. There were extremely significant positive correlations between sediment concentration, sediment yield, and Cd, Cu, As, Pb, and Zn loss. Runoff and coverage were correlated with PHMs (Mantel’s r were 0.24 and 0.12, respectively, *p* < 0.05), indicating that runoff and coverage affect the loss form of trace elements.

The structural equation model (SEM) was used to assess the effects of rainfall, ecological buffer width, and vegetation coverage on trace element loss (Figure 6). Apart from the direct positive effect of rainfall on trace element loss, indirect positive effects of rainfall on trace element loss through runoff and sediment concentration were observed. Unlike the effect of rainfall, the effects of riparian zone width and coverage were indirectly negatively correlated with trace element loss through the runoff and sediment concentration pathways and the sediment concentration pathway, respectively.

## 4. Discussion

In this study, the loss of sediment and trace elements from riparian zones was observed primarily during low coverage and heavy rainfall periods, which was similar to farmland and meadows [27,28]. Studies focused on riparian zones within 10 m found a loss control effect of 10%–98% for nitrogen, phosphorus, and pesticides. In comparison, a higher efficiency of 41%–99% was observed for trace element removal by runoff in this study [29,30]. The observation periods in this study were brief, so the long-term effectiveness of riparian zones in removing trace elements from runoff remains to be determined.

In mining wastelands, trace elements migrate primarily with sediment due to the erodible soil. The loss of soil and trace elements in the experimental field was comparable to that in the manganese tailings area [31]. The concentrations of trace elements in sediment and dissolved trace elements in runoff were closely correlated with the content and solubility of trace elements in the soil [32]. Organic matter alters the surface properties of soil particles, diminishing the internal repulsive force and enhancing the stability and erosion resistance of soil aggregates [33]. Lack of organic matter and vegetation cover make the experiment field more erodible compared to farmland and riparian areas [34]. The concentrations of trace elements in sediment were found to be correlated with rainfall, coverage, and sediment concentration based on correlation analysis. Trace element concentrations and P_HMs_ increased during periods of heavy rainfall and on bare land. Soil particles enriched with trace elements are transported via splashing and erosion [31,35,36]. In mining wastelands, trace elements are primarily lost in sediments on bare grounds and during periods of heavy rainfall.

Vegetation coverage in the riparian zone reduces soil and trace element loss. In ryegrass-covered soil, sediment yield decreased as vegetation coverage increased, and increased with rainfall intensity [37]. Correlation analysis revealed a significant relationship between coverage and the concentrations of sediment and trace elements. Additionally, SEM analysis demonstrated that coverage had a negative effect on sediment concentration and trace element loss. *R. simsii* and *L. perenne* buffered the erosion from rainfall and reduced the splash distance of the soil and the amount of particle stripping [38]. The increase in surface biomass, particularly the rise in plant stem density and roughness, improved filtration and capture effectiveness of soil particulates and decreased sediment and trace element concentrations [39]. The intercropping of *R. simsii* and *L. perenne* constructed an intertwined shallow root system in the soil [40], formed biological pores and soil cracks, reduced soil compactness, and improved the permeability and water storage capacity of the soil [41]. Therefore, plant-based soil stabilization and structural improvement are important mechanisms for reducing trace element loss [42].

Shrubs and herbaceous plants exhibit different abilities in reducing sediment and trace element loss. For instance, the plant canopy of *R. simsii* intercepts rainfall, resulting in the formation of streamflow and a reduction in effective rainfall [43]. With its high leaf and stem density per unit area, *L. perenne* increases hydraulic resistance and reduces runoff. As a result, these plant characteristics enhance the critical hydrodynamic force for initiating soil erosion. Intercropping patterns proved effective in controlling soil erosion on slopes [44]. A study showed that the *R. simsii* / *L. perenne* community had a more effective stabilizing effect on trace elements than an *L. perenne* riparian community alone [24].

A wide riparian zone reduced the loss of runoff and trace elements. Compared with the 5 m riparian zone, a double length of the plant filter network and soil porosity along the direction of water flow were constructed in the 10 m riparian zone. *R. simsii* and *L. perenne* in the wider riparian zone slowed water flow and enhanced water retention and infiltration in the riparian zone. As a result, less water flowed out of the riparian zone. Studies have shown that riparian zone width and infiltration rate have no influence on sediment concentration, as the sediment remains suspended over a certain width [18,45,46]. Correlation analysis and SEM analysis indicated that the width of the buffer zone was not correlated with sediment and trace element concentrations. The wide riparian zone enhanced the water retention capacity of the soil, reduced runoff, and reduced the loss of sediment and trace elements. However, inconsistent results were observed during heavy rainfall periods.

Vegetation protection of the soil decreased during heavy rainfall periods [38]. Sediment concentration and yield increased with the increase in rainfall kinetic energy, even when the surface coverage reached 80% [39,47]. The loss and concentrations of sediment and trace elements in the 10 m riparian zone were significantly lower than in the 5 m riparian zone. Multivariate analysis indicated that there was an interaction between rainfall intensity and riparian zone width, which affected the loss and concentrations of sediment and trace elements. During heavy rainfall periods, a wide plant filtration network adsorbed soil particulates and promoted sediment and trace element precipitation. Thus, the impact of the mechanism of the riparian zone width on trace element loss existed under various scenarios: The wide riparian zone reduced runoff during normal rainfall periods and reduced trace element concentrations and runoff during heavy rainfall periods.

Buffer zone width affected the loss forms of trace elements. The loss forms of trace elements were found to be correlated with the forms of trace elements in soil, solubility in water, sediment carriers, and rainfall intensity [48,49]. With an increase in riparian zone width, the concentration of trace elements in sediment and dissolved Cd decreased, while the concentration of dissolved As increased. There was a nonlinear relationship between riparian zone width and trace element removal efficiency [50]. Hydraulic retention duration may influence the concentration of dissolved trace elements [51].

Vegetation coverage stabilized trace elements in the riparian zone. *R. simsii* and *L. perenne* showed potential in phytoextraction for soils contaminated with trace elements [52,53]. In the intercropping mode, the organic acids secreted by *R. simsii* roots may increase the bioavailability of trace elements in the soil, enhance the ability of *R. simsii* and *L. perenne* to accumulate trace elements, and promote the accumulation of *L. perenne* biomass [54,55]. This intercropping mode facilitated the transfer of trace elements in the soil to the vegetation, which, in turn, reduced the loss of trace elements from the riparian zone [9].

## 5. Conclusions

Trace element loss in the riparian zone was primarily observed during low coverage and heavy rainfall periods. The construction of the *R. simsii*/*L. perenne* riparian zone increased vegetation coverage, promoted water infiltration, and captured and filtered soil particles in runoff. Compared to the 5 m riparian zone, sediment and trace element loss in the 10 m riparian zone decreased during heavy rain periods due to a reduction in runoff yield and a reduction in sediment and trace element concentrations in the runoff. The effects of riparian zone width were directly negatively correlated with runoff and indirectly negatively affected by sediment concentration and trace element loss. Furthermore, trace element loss was sensitive to vegetation coverage during low coverage periods, and sensitive to riparian zone width during high coverage periods. In general, constructing a 10 m riparian zone is an effective measure for controlling the loss of trace elements in mining wastelands, although the long-term effects remain to be determined.

## Figures and Tables

**Table 1:** Amounts of biomass and trace elements accumulated in plants in the riparian zones.

Plant		Biomass (kg m^−2^)	Cd (mg m^−2^)	Cu (mg m^−2^)	As (mg m^−2^)	Pb (mg m^−2^)	Zn (mg m^−2^)
*Lolium perenne* L.	Shoot	0.44 ± 0.15 a	1.71 ± 0.83 a	2.58 ± 1.27 b	0.60 ± 0.40 a	37.94 ± 22.71 a	198.83 ± 111.04 a
	Root	0.01 ± 0.01 d	1.02 ± 0.44 b	0.50 ± 0.22 c	0.68 ± 0.38 a	37.47 ± 25.87 a	110.68 ± 74.92 b
*Rhododendron simsii*	Shoot	0.10 ± 0.01 c	0.29 ± 0.04 c	0.84 ± 0.18 c	0.19 ± 0.05 b	13.96 ± 1.84 b	21.58 ± 11.15 c
	Root	0.16 ± 0.05 b	0.26 ± 0.09 c	3.45 ± 0.87 a	0.10 ± 0.04 b	10.66 ± 2.56 b	18.61 ± 6.20 c

Note: The different lowercase letters mean significant differences in biomass and accumulated trace elements between different plant parts at the *p* < 0.05 level.

## Data Availability

The data presented in this study are available upon request from the corresponding author.

## Supplementary Materials

The following supporting information can be downloaded at: https://www.mdpi.com/article/10.3390/toxics12040279/s1, Figure S1: rainfall characteristics and riparian zone coverage, Figure S2: pH of runoff, Figure S3: Scatter diagram of principal component analysis.

## References

[1] Ali H., Khan E., Ilahi I. (2019). Environmental Chemistry and Ecotoxicology of Hazardous Heavy Metals: Environmental Persistence, Toxicity, and Bioaccumulation. J. Chem..

[2] Ding Q., Cheng G., Wang Y., Zhuang D. (2017). Effects of natural factors on the spatial distribution of heavy metals in soils surrounding mining regions. Sci. Total Environ..

[3] Žibret G., Gosar M., Miler M., Alijagić J. (2018). Impacts of mining and smelting activities on environment and landscape degradation—Slovenian case studies. Land Degrad. Dev..

[4] Li B., Deng J., Li Z., Chen J., Zhan F., He Y., He L., Li Y. (2022). Contamination and health risk assessment of heavy metals in soil and ditch sediments in long-term mine wastes area. Toxics.

[5] Chen L., Zhang H., Xie Z., Ding M., Devlin A.T., Jiang Y., Xie K. (2022). The temporal response of dissolved heavy metals to landscape indices in the Le’an river, China. Environ. Res..

[6] Chen J.Q. (1996). Riparian vegetation characteristics and their functions in ecosystems and landscapes. Chin. J. Appl. Ecol..

[7] Liu X., Zhang X., Zhang M. (2008). Major Factors Influencing the Efficacy of Vegetated Buffers on Sediment Trapping: A Review and Analysis. J. Environ. Qual..

[8] Borin M., Passoni M., Thiene M., Tempesta T. (2010). Multiple functions of buffer strips in farming areas. Eur. J. Agron..

[9] Sun C., Liu H.W., Gong L.F., Cao X.P., Shen H.L., Gao D.W. (2019). Purification of Three Herbal Buffer Strips for Non-point Source Pollution of Copper and Lead. Chin. J. Soil Sci..

[10] Wang J., Luo X.H., Zhang Y.F., Huang Y.H., Rajendran M., Xue S.G. (2018). Plant species diversity for vegetation restoration in manganese tailing wasteland. Environ. Sci. Pollut. Res. Int..

[11] Tang J.X., He M.M., Wang D.H., Zeng X.F., Li R.J., Ying B. (2016). Suspended sediments and runoff reduction by established riparian vegetated filter strips. Chin. J. Environ. Eng..

[12] Fu J., Wang Y.Q., Ma C., Wang Y.J., Liang D. (2019). Research Progress on the Effects of Vegetation Buffer Zone on Reducing Agricultural Non-point Pollution. J. Soil Water Conserv..

[13] McMahon J.M., Olley J.M., Brooks A.P., Smart J.C.R., Stewart-Koster B., Venables W.N., Curwen G., Kemp J., Stewartd M., Saxton N. (2020). Vegetation and longitudinal coarse sediment connectivity affect the ability of ecosystem restoration to reduce riverbank erosion and turbidity in drinking water. Sci. Total Environ..

[14] Ouyang W., Wang Y., Lin C., He M., Hao F., Liu H., Zhu W. (2018). Heavy metal loss from agricultural watershed to aquatic system: A scientometrics review. Sci. Total Environ..

[15] Li J.G., Li Z.X., Brandis K.J., Bu J.W., Sun Z.Y., Yu Q., Ramp D. (2020). Tracing geochemical pollutants in stream water and soil from mining activity in an alpine catchment. Chemosphere.

[16] Chen D., Chen Y.P., Lin Y.S. (2021). Heavy rainfall events following the dry season elevate metal contamination in mining-impacted rivers: A case study of wenyu river, Qinling, China. Arch. Environ. Contam. Toxicol..

[17] Chen Z., Liu X., Ai Y., Chen J., Luo X., Chen J., Zhong S. (2018). Effects and mechanisms of revegetation modes on cadmium and lead pollution in artificial soil on railway rock-cut slopes. Sci. Total Environ..

[18] Miller J.J., Curtis T., Chanasyk D.S., Reedyk S. (2015). Influence of mowing and narrow grass buffer widths on reductions in sediment, nutrients, and bacteria in surface runoff. Can. J. Soil Sci..

[19] Zhou J., Fu B., Gao G., Lü Y., Liu Y., Lü N., Wang S. (2016). Effects of precipitation and restoration vegetation on soil erosion in a semi-arid environment in the Loess Plateau, China. Catena.

[20] Rao Z.X., Huang D.Y., Zhu H.H., Zhu Q.H., Wang J.Y., Luo Z.C., Xu C., Shen X., He Y.B. (2016). Effect of rice straw mulching on migration and transportation of Cd, Cu, Zn, and Ni in surface runoff under simulated rainfall. J. Soils Sediments.

[21] King S.E., Osmond D.L., Smith J., Burchell M.R., Dukes M., Evans R.O., Knies S., Kunickis S. (2016). Effects of Riparian Buffer Vegetation and Width: A 12-Year Longitudinal Study. J. Environ. Qual..

[22] Hao R.X., Xue L.M., Liu R.X., Sun F., Li X.J., Yuan P., Zhou Y.M. (2022). VFSMOD model-based simulation of interception by ecological buffer zone under different scenarios. Chin. J. Environ. Eng..

[23] Xu H.S., Li C.Y., Wen C., Zhu S.J., Zhu S.Q., Li N.H., Li R.F., Luo X. (2023). Heavy metal fraction, pollution, and source-oriented risk assessment in biofilms on a river system polluted by mining activities. Chemosphere.

[24] Deng J., Zhang C., Li Y., Li B., Zu Y., Li Y., Chen J., Zhang Z. (2022). Effect of ecological buffer patterns on abating soil erosion and heavy metal diffusion in riverbanks near mining waste catchments. J. Agro-Environ. Sci..

[25] Zhiyuan W., Dengfeng W., Huiping Z., Zhiping Q. (2011). Assessment of Soil Heavy Metal Pollution with Principal Component Analysis and Geoaccumulation Index. Procedia Environ. Sci..

[26] Huang J., Wang C., Qi L., Zhang X., Tang G., Li L., Guo J., Jia Y., Dou X., Lu M. (2020). Phosphorus is more effective than nitrogen in restoring plant communities of heavy metals polluted soils. Environ. Pollut..

[27] Zuazo V.H.D., Pleguezuelo C.R.R., Peinado F.J.M., de Graaff J., Martínez J.R.F., Flanagan D.C. (2011). Environmental impact of introducing plant covers in the taluses of terraces: Implications for mitigating agricultural soil erosion and runoff. CATENA.

[28] Niu Y., Li S., Liu Y., Shi J., Wang Y., Ma Y., Wu G.-L. (2021). Regulation of alpine meadow patch coverage on runoff and sediment under natural rainfall on the eastern Qinghai-Tibetan Plateau. J. Hydrol..

[29] Gene S.M., Hoekstra P.F., Hannam C., White M., Truman C., Hanson M.L., Prosser R.S. (2019). The role of vegetated buffers in agriculture and their regulation across Canada and the United States. J. Environ. Manag..

[30] Prosser R.S., Hoekstra P.F., Gene S., Truman C., White M., Hanson M.L. (2020). A review of the effectiveness of vegetated buffers to mitigate pesticide and nutrient transport into surface waters from agricultural areas. J. Environ. Manag..

[31] Wang J., Cheng Q.Y., Xue S.G., Rajendran M., Wu C., Liao J.X. (2018). Pollution characteristics of surface runoff under different restoration types in manganese tailing wasteland. Environ. Sci. Pollut. Res..

[32] Wei L.H., Liu Y., Routh J., Tang J.F., Liu G.W., Liu L.R., Luo D.G., Li H.S., Zhang H.G. (2019). Release of heavy metals and metalloids from two contaminated soils to surface runoff in southern china: A simulated-rainfall experiment. Water.

[33] Ma R., Hu F., Xu C., Liu J., Zhao S. (2022). Response of soil aggregate stability and splash erosion to different breakdown mechanisms along natural vegetation restoration. CATENA.

[34] Smith D.J., Snead M., Thompson T.M. (2022). Soil amended with organic matter increases fluvial erosion resistance of cohesive streambank soil. J. Geophys. Res. Biogeosci..

[35] Wu T., Li X.P., Cai Y., Ai Y.W., Sun X.M., Yu H.T. (2017). Geochemical behavior and risk of heavy metals in different size lead-polluted soil particles. China Environ. Sci..

[36] Xu C.Y., Zhou T.T., Wang C.L., Liu H.Y., Zhang C.T., Hu F.N., Zhao S.W., Geng Z.C. (2020). Aggregation of polydisperse soil colloidal particles: Dependence of Hamaker constant on particle size. Geoderma.

[37] Dong Y., Lei T., Li S., Yuan C., Zhou S., Yang X. (2015). Effects of ryegrass coverage on soil loss from loess slopes. Int. Soil Water Conserv. Res..

[38] Yao J.J., Cheng J.H., Zhou Z.D., Sun L., Zhang H.J. (2018). Effects of herbaceous vegetation coverage and rainfall intensity on splash characteristics in northern China. CATENA.

[39] Pan D., Gao X., Dyck M., Song Y., Wu P., Zhao X. (2017). Dynamics of runoff and sediment trapping performance of vegetative filter strips: Run-on experiments and modeling. Sci. Total Environ..

[40] Reubens B., Poesen J., Danjon F., Geudens G., Muys B. (2007). The role of fine and coarse roots in shallow slope stability and soil erosion control with a focus on root system architecture: A review. Trees.

[41] Hao H.X., Wei Y.J., Cao D.N., Guo Z.L., Shi Z.H. (2020). Vegetation restoration and fine roots promote soil infiltrability in heavy-textured soils. Soil Tillage Res..

[42] Löbmann M.T., Geitner C., Wellstein C., Zerbe S. (2020). The influence of herbaceous vegetation on slope stability—A review. Earth-Sci. Rev..

[43] Zwieback S., Chang Q., Marsh P., Berg A. (2019). Shrub tundra ecohydrology: Rainfall interception is a major component of the water balance. Environ. Res. Lett..

[44] Li X., Zhang Y., Ji X., Strauss P., Zhang Z. (2022). Effects of shrub-grass cover on the hillslope overland flow and soil erosion under simulated rainfall. Environ. Res..

[45] Schmitt T.J., Dosskey M.G., Hoagland K.D. (1999). Filter Strip Performance and Processes for Different Vegetation, Widths, and Contaminants. J. Environ. Qual..

[46] Wei Y., Wu X., Xia J., Zeng R., Cai C., Wang T. (2019). Dynamic study of infiltration rate for soils with varying degrees of degradation by water erosion. Int. Soil Water Conserv. Res..

[47] Liu Q., Deng D., Liao Q., Ying B. (2021). Analysis on the influence of rainfall characteristics on soil and water loss in rocky desertification region. Carbonates Evaporites.

[48] Gaiero D., Probst J., Depetris P., Lelyter L., Kempe S. (2002). Riverine transfer of heavy metals from Patagonia to the southwestern Atlantic Ocean. Reg. Environ. Chang..

[49] Liu M., Fan D., Bi N., Sun X., Tian Y. (2019). Impact of water-sediment regulation on the transport of heavy metals from the Yellow River to the sea in 2015. Sci. Total Environ..

[50] Lv J., Wu Y. (2021). Nitrogen removal by different riparian vegetation buffer strips with different stand densities and widths. Water Supply.

[51] Zhou B.B., Chen X.P., Su L.J., Li H.J., Wang Q.J., Tao W.H. (2021). Evaluation and modeling of factors influencing the depth of mixing layer in which soil solute releasing from soil to surface runoff. Can. J. Soil Sci..

[52] Chang L.X., Liu F.S., Zhan F.D., Li B., Chen J.J., Zu Y.Q., Li Y., He Y.M. (2023). Growth adaptability of 13 *Rhododendron* varieties in complex polluted cropland in a plateau lead-zinc mining area. Chin. J. Ecol..

[53] Zhang J., Yang N.N., Geng Y.N., Zhou J.H., Lei J. (2019). Effects of the combined pollution of cadmium, lead and zinc on the phytoextraction efficiency of ryegrass (*Lolium perenne* L.). RSC Adv..

[54] Xu T.Y., Quan W.X., Li C.C., Pan Y.N., Xie L.J., Hao J.T., Gao Y.D. (2021). Distribution characteristics of low molecular weight organic acids in soil of wild rhododendron forest. Sci. Silvae Sin..

[55] Zou W., Cao Z., Wang Y., Jin M., Lin M. (2022). Intercropping of *Pennisetum sinese* with *Lolium perenne* improved phytoextraction of heavy metal from soil. Restor. Ecol..

