# Supplementary material for: Wide Riparian Zones Inhibited Trace Element Loss in Mining Wastelands by Reducing Surface Runoff and Trace Elements in Sediment
Source: Toxics. 2024 Apr 11;12(4):279. doi: 10.3390/toxics12040279 (PMC11053404; doi:10.3390/toxics12040279)
Supplement: Supplementary file 1 [file toxics-12-00279-s001.zip › toxics-2924350-supplementary.pdf]

toxics

Article

Wide Riparian Zones Inhibited Trace Element Loss in Mining Wastelands by Reducing Surface Runoff and Trace Elements in Sediment

Jiangdi Deng [1,†], Zuran Li [2,†], Bo Li [3], Cui Xu [1], Lei Wang [3] and Yuan Li [3,*]

[1] Faculty of Animal Science and Technology, Yunnan Agricultural University, Kunming 650201, China; dengjd.cn@gmail.com (J.D.); cuixu0415@126.com (C.X.)
[2] College of Horticulture and Landscape, Yunnan Agricultural University, Kunming 650201, China; lizuran@foxmail.com
[3] College of Resources and Environment, Yunnan Agricultural University, Kunming 650201, China; libo@ynau.edu.cn (B.L.); wanglei.ko@foxmail.com (L.W.)
* Correspondence: liyuan@ynau.edu.cn
† These authors contributed equally to this work.

Citation: Deng, J.; Li, Z.; Li, B.; Xu, C.; Wang, L.; Li, Y. Wide Riparian Zones Inhibited Trace Element Loss in Mining Wastelands by Reducing Surface Runoff and Trace Elements in Sediment. *Toxics* **2024**, *12*, x. https://doi.org/10.3390/xxxxx

Academic Editor: Ezio Ranieri

Received: 4 March 2024
Revised: 5 April 2024
Accepted: 8 April 2024
Published: date

Copyright: © 2024 by the authors. Licensee MDPI, Basel, Switzerland. This article is an open access article distributed under the terms and conditions of the Creative Commons Attribution (CC BY) license (https://creativecommons.org/licenses/by/4.0/).

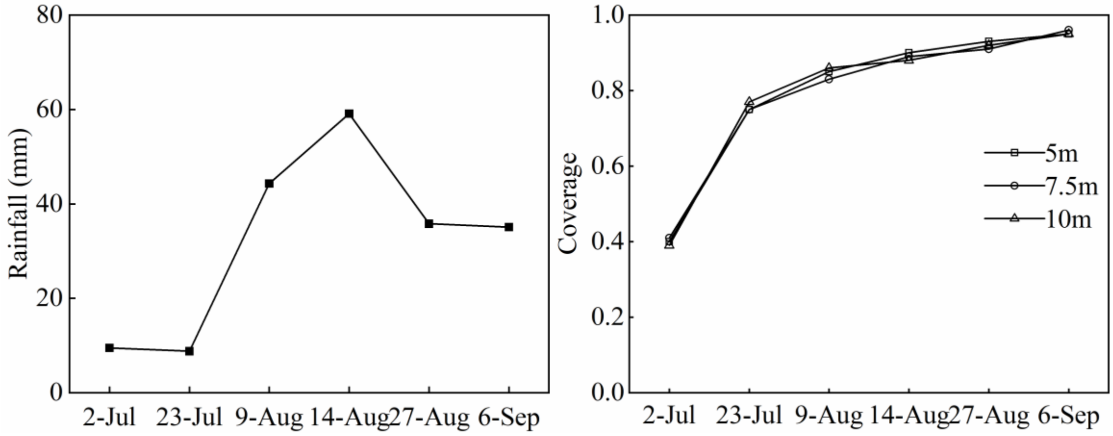

Figure S1. Rainfall characteristics and riparian zone coverage.

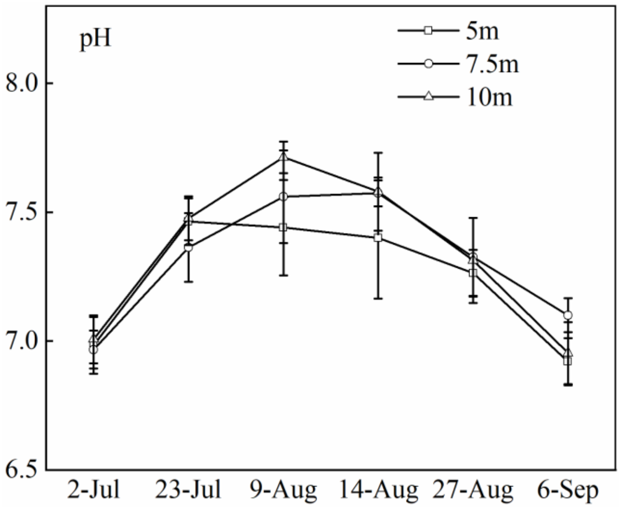

Figure S2. pH of runoff.

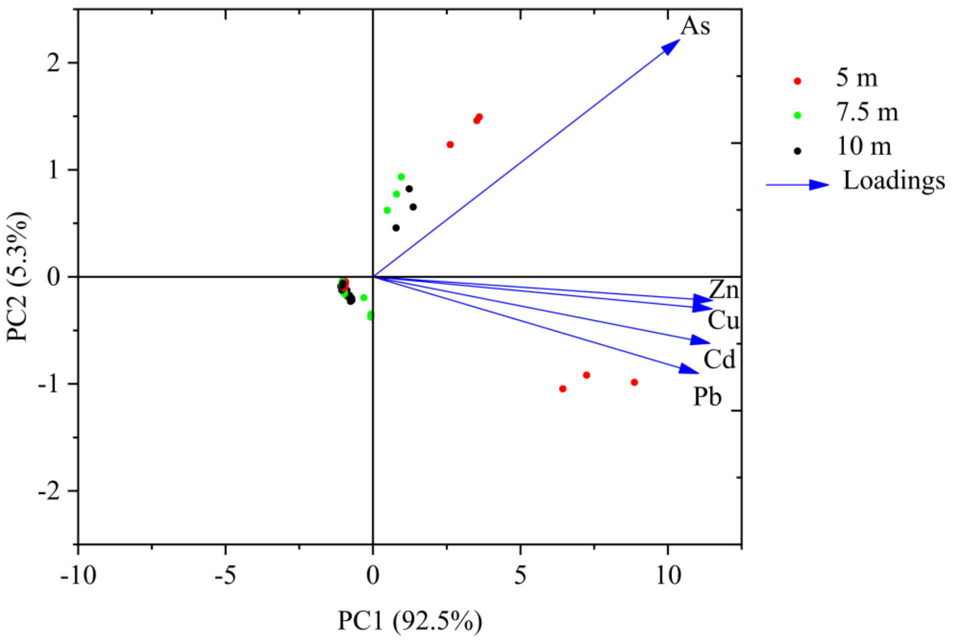

Figure S3. Scatter diagram of principal component analysis.

Disclaimer/Publisher's Note: The statements, opinions and data contained in all publications are solely those of the individual author(s) and contributor(s) and not of MDPI and/or the editor(s). MDPI and/or the editor(s) disclaim responsibility for any injury to people or property resulting from any ideas, methods, instructions or products referred to in the content.
